# Effect of emergence profile and space gap size on excess cement in cement-retained implant reconstructions

**DOI:** 10.34172/japid.025.2292

**Published:** 2025-04-21

**Authors:** Fariborz Vafaee, Saeed Nikanjam, Arash Farahnaki, Meysam Mahabadi, Sajjad Farashi, Sara Khazaei, Shiva Shahabi

**Affiliations:** ^1^Dental Implants Research Center, Avicenna Institute of Clinical Sciences, Avicenna Health Research Institute, Hamadan University of Medical Sciences, Hamadan, Iran; ^2^Department of Prosthodontics, School of Dentistry, Hamadan University of Medical Sciences, Hamadan, Iran; ^3^Department of Prosthodontics, School of Dentistry, Islamic Azad University, Isfahan Branch (Khorasgan), Isfahan, Iran; ^4^Neurophysiology Research Center, Institute of Neuroscience and Mental Health, Avicenna Health Research Institute, Hamadan University of Medical Sciences, Hamadan, Iran

**Keywords:** Cementation, Dentistry, Emergence profile, Gap, Implant

## Abstract

**Background.:**

Excess cement is one of the most prevalent problems with cement-retained implant-supported prostheses. The excess cement may be considered an important source of inflammation during implant application. Optimizing the design procedure for reducing excess cement is necessary for successful and safe implant applications.

**Methods.:**

This study assessed the effect of two confounding factors, i.e., emergence profile and space gap size, on the level of excess cement. Three types of emergence profiles (concave, convex, and straight) and three different gap sizes (30, 60, and 90 µm) were considered for implant design, and the level of excess cement was measured for each design.

**Results.:**

Statistical analyses using one-way ANOVA followed by post hoc *P* value correction revealed that the best emergence profile with the lowest excess cement was the straight profile, with statistically lower excess cement compared with concave and convex profiles (*P*<0.05) and no significant difference between concave and convex profiles. Furthermore, analyses showed that lower gap size was associated with lower excess cement, even though increasing the gap size from a threshold (>60 µm) made the difference significant. Interaction analysis using two-way ANOVA also indicated the interaction between the emergence profile and space gap size.

**Conclusion.:**

The results emphasized that a straight profile with a smaller gap size should be considered to reduce the excess cement. However, due to the small sample size of the study, further analyses with different types of materials, angles of deformation, and gap sizes are required to reveal the exact relationship between excess cement and the design specifications.

## Introduction

 The dental implant attachment system for oral restorations should be specified before the surgery since design parameters profoundly affect the quality of the sample.^1^ Previous studies showed that both dental cement-retained and screw-retained implant reconstruction systems were successfully applied in real applications (success rates of 89.3% and 96.5% for screw-retained and cement-retained attachment systems, respectively).^2^ There are numerous advantages to the application of cement-retained implant restorations, including compensation of incorrectly inclined implants, easier passive fit, easier control of occlusion,^3^ the ease of splinting implant, reduced incidence of prosthesis detachment, ease of fabrication and cost, low incidence of retention and so on.^4^

 The most prevalent problem with cement-retained implant-supported prostheses is the remaining cement *in the gingival sulcus‒implant* interface,^5^ which is a source of inflammation in the surrounding areas of the implant.^6,7^ In other words, the excess cement acts like an artificial calculus and endangers the health of dental implants and adjacent soft tissues.^8,9^ Considering the excess cement, different confounding factors such as the depth of the prosthesis margin, the emergence profile (convex or concave), the used cement type and cementing technique, the type and height of abutment, and the size of space of the abutment screw hole access were studied in the previous researches.^10^

 Excess cement is one of the risk factors for the accumulation of plaques at implants and the increased risk of peri-implantitis,^8^ especially in patients with a history of periodontal disease.^11^ The remaining cement may also be associated with bleeding and inflammation.^12^ In this regard, excess residual cement is a significant concern when attaching the implant to the supported restorations.^13^

 Several studies investigated how cementation techniques in implant restorations affect the excess cement level. Canullo et al^14^ compared two different intraoral and extraoral modalities of implant-supported cementation on excess cement. This study showed that the extraoral cementation outperformed the intraoral modality in the case of excess cement. Another study investigated the depth of the cementation margin on the cement remnants on implants. The results showed that reducing the undercuts helped better remove cement excess.^15^ This result was not dependent on the diameter and location of the implant in the oral cavity. Frisch et al^16^ proposed an extraoral replica technique for minimizing excess cement. This study concluded that the zinc oxide cement provided adequate retention for implant-supported restorations. Vafaee et al^17^ compared excess cement at the marginal area of implant-supported crowns between three different techniques (i.e., PTEE tape, replica, and conventional techniques). The results confirmed that the cementation technique was an important factor in the amount of excess cement. Gehrke et al^18^ investigated the effect of margin location and material on excess cement of implant abutments that were created using CAD/CAM systems. The results revealed that excess cement depended on the crown-abutment margin, and the maximal excess cement was obtained for more submucosally cases.

 Al Amri et al^19^ investigated the effects of three different types of abutment replicas on the excess cement in crown marginal areas. According to this study, the lowest remaining cement was related to the abutment replica produced by the pattern resin (3D-printed) analog technique. The space size of the abutment screw access channel was also shown to be effective on the amount of excess cement.^19^ According to Al Amri et al,^19^ by increasing the marginal space to 2 mm, the amount of excess cement was reduced by 55% compared to the nonspace model; however, the marginal accuracy was not significantly affected by the space size. This was confirmed by a study by Linkevicius et al,^7^ in which the highest excess cement level was observed for restoration margins located more subgingivally. Liang et al^13^ tested three techniques for cementing crown to the implant‒abutment complex consisting of evenly placing cement and removal of excess cement by an explorer, using a small amount of cement without excess cement removal and using a large amount of cement and removal process using a resin abutment replica. This study showed that the resin abutment replica reduced the excess cement significantly.^13^ The emergence profile of the abutment also affected the excess cement.^20^ It was shown that the concave emergence profile abutments significantly increased the risk of cement excess compared to a convex model.^20^ With deeper crown‒abutment margin positions, the risk of excess cement was also increased.^20^ Patel et al^21^ showed that the presence of a vent hole could affect the excess cement. Furthermore, Chee et al^12^ showed that the amount of excess cement decreased when the excess cement was displaced before the seating of the crown on the abutment.

 There are several unresolved questions regarding the association between gap size in the design of the abutment replica or the emergence profile design and the excess cement in cement-retained implant restorations. A few studies have been performed so far to find such an association. Here, we hypothesized that emergence profile and gap size might be two important confounding factors in the excess cement in the neighboring tissues of cement-retained implants. Furthermore, the association of these factors was also investigated.

## Methods

###  Preparation of customized abutments

 An anonymous clinical case (patient) treated with cement-retained implant-supported prostheses at the central incisor was selected from the Department of Prosthodontics, Hamadan Dental School. The inserted implant dimension was 11.5 × 4.2 and contained an internal connection (SIC invent AG; Birmannsgasse 3, BASEL-STADT, 4055, Switzerland(. Before casting, the soft tissue around the implant was modeled to an ideal shape using a temporary restoration; then, using the open tray technique to record the emergence profile accurately, it was transferred to the final cast using a customized impression coping. The final cast was prepared using *type IV gypsum product, and soft tissue was reconstructed using *the gingival mask(G-mask; Coltène/Whaledent GmbH, Germany). In the current experimental study, three customized abutments with concave, convex, and straight emergence profiles were designed using CAD software (Exocad DentalCAD; Exocad GmbH Inc., Germany). The marginal depth of restoration was 2 mm in all areas and the customized gingival masks were used for each type of abutment.

**Figure 1:**
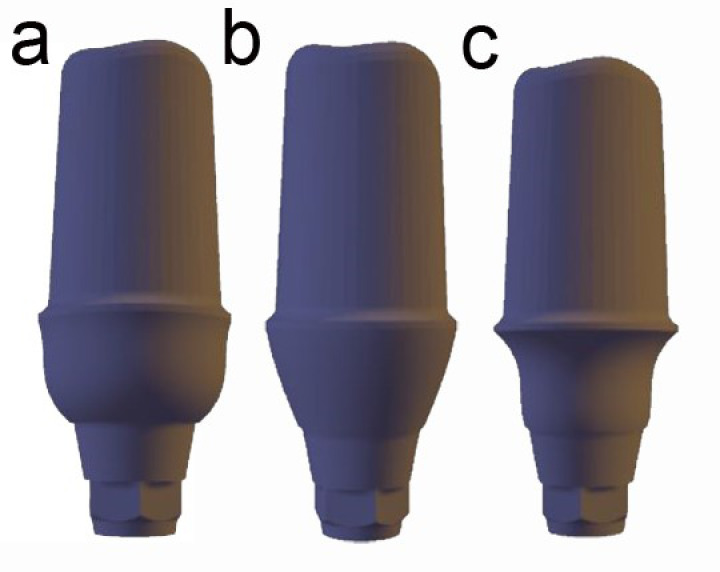
Three emergence profile types: (a) concave, (b) straight, and (c) convex

 The stereolithography (STL) file (Figure 1) for the designed abutments was used by a 3D printer (Asiga Freeform 2; Asiga, Alexandria, Australia) to produce resin-based models of abutments. The 3D printing support structures were added to the abutment models (oriented at 45º compared with the printer build platform) using the associated software Asiga Composer (Asiga, Alexandria, Australia, Version 1.2). The 3D-printed resin-based abutments were subjected to a standardized casting protocol using Cobalt chromium alloy (Colado CC; Ivoclar Vivadent, Liechtenstein).

###  Preparation of abutment replicas

 In this study, three models of abutment replicas with different gap sizes (30, 60, and 90 µm) were designed and printed using a 3D printer.

###  Preparation of restorations 

 For each designed abutment, a temporary resin-based restoration was designed using CAD/CAM technology (Vita cad temp; VITA Zahnfabrik H. Rauter GmbH & Co.KG, Germany). The color for restoration was M2.

###  Cementing restorations

 A phantom head was used for the cementing procedure to mimic the clinical situation. The phantom head was positioned so that the occlusal plane was perpendicular to the floor, with the dentist in the 10 O’clock position. The cementation was performed by an independent dentist who was not aware of the aim and scope of the study. For monolithic restorations, zinc oxide eugenol* cement*was used.After cement mixing, a thin layer of cement was placed on the marginal area of restoration, and the restoration was placed on the intended replica. The cementation procedure was terminated by placing the replica on the implant’s abutment. The exerted force on restoration was preserved for 10 minutes until the final setting.

###  Removing excess cement

 After the final cement setting, another dentist who was not familiar with the aim and scope of the current study removed the excess cement. A periapical radiographic image was used to ensure the removal of excess cement.

###  Measuring excess cement

 The restoration was separated from the analog implant using an access cavity on the palatal surface.The remaining particles of eugenol zinc oxide cement were measured using a digital balance** (**BL120 Sartorius; Germany) with a measurement accuracy of 0.0001 g.

###  Sample size estimation

 The required sample size was calculated according to the following formula (Equation 1)^22^:

 (1)

$$n=\frac{(\sigma_{1}^{2\;}\;+\sigma_{2}^{2})\;{\left[Z_{1-\frac{\alpha }{2}}+\;Z_{1-\beta }\right]}^{2}}{{\left(\mu_{1}\;-\;\mu_{2}\right)}^{2}}$$

in which, *σ*_i_ and *μ*_i_ are the variance and the mean value for the *i*-*th* group obtained from a previous study.^20^ For calculating the sample size of the study, the mean difference was adjusted to 0.9, and the significance level (α) and the statistical power (1-β) were adjusted to 0.05 and 80%, respectively, determining at least 6 samples in each group. We used 8 samples for each category (i.e., convex/gap: 30 µm, convex/gap: 60 µm, convex/gap: 90 µm, concave/gap: 30 µm, concave/gap: 60 µm, concave/gap: 90 µm, *straight*/gap: 30 µm, *straight*/gap: 60 µm, *straight*/gap: 90 µm).

###  Statistical analysis

 The normality test for excess cement data for each condition (different emergence profiles and different gap sizes) was performed using the Kolmogorov-Smirnov test.^23^ When there was insufficient evidence to reject the null hypothesis of normal distribution, one-way and two-way ANOVA were performed. For one-way ANOVA, the confounding factors were the type of emergence profile (concave, convex, and straight) and the gap size (30, 60, and 90 µm). The interaction between these two factors was also evaluated using a two-way ANOVA. The post hoc Tukey-Kramer *P *value correction method was applied to the result of the ANOVA analysis to correct the *P *value for multiple comparison analyses. For all statistical analyses, the significance level of 0.05 was used. In the case of non-normal distribution, non-parametric Mann-Whitney tests were used.

 The mean difference between groups was also calculated using Hedge’s g formula as follows:

 (2)


$$Hedge'sg=\;\frac{M_{1}\;-M_{2}}{\sqrt{\frac{SD_{1}^{2}\;+\;SD_{2}^{2}}{2}}}\;\left[1\;-\;\frac{3}{4(n_{1}\;+\;\;n_{2})\;-\;9}\right]$$

in which *M*_i_, *SD*_i_, and *n*_i_ represent the mean, standard deviation, and sample size for *the i-th* group, respectively. Furthermore, for descriptive analyses, mean ± SD was used (SD stands for standard deviation). All analyses were performed using MATLAB (MathWorks Inc, USA, MA, version 2017) and its statistical toolbox.

## Results

Table 1 shows the values for excess cement (gr) of the cement-retained implant reconstructions. These values were obtained with different emergence profiles (concave, convex, and straight) and different margin sizes (30, 60, and 90 µm) for eight independent samples.

 The standardized mean difference (Hedge’s g) between excess cement for different designs was calculated and reported (Table 2). The mean difference shows how excess cement changes when the design specifications are altered from the values specified in the left column of Table 2 (group 1) to those specified in the first row of Table 2 (group 2). A negative value indicates increased excess cement.

**Figure 2:**
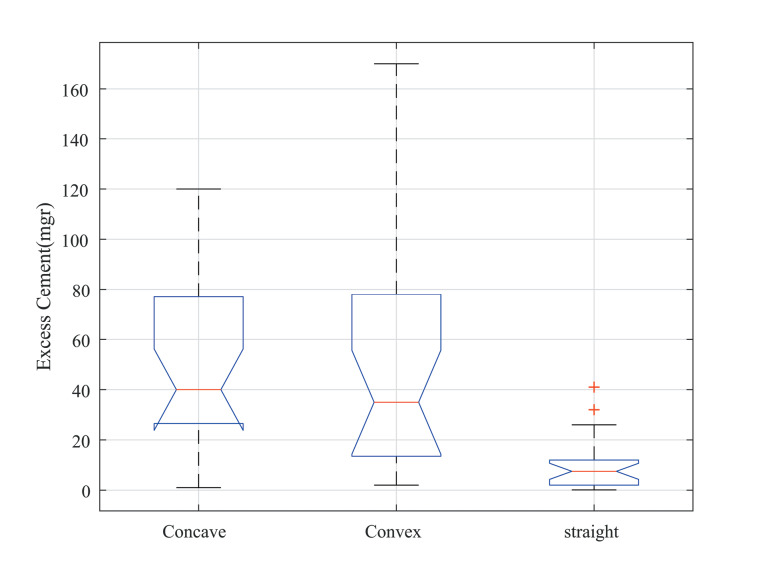
Average excess cement (mg) for different emergence profiles

 The one-sample Kolmogorov-Smirnov normality test showed that all data (column of Table 1) came from a standard normal distribution (Table 2). In this regard, ANOVA was used for further analyses. Considering the emergence profile, the statistical analysis using one-way ANOVA showed significant differences between the three groups (i.e., convex, concave, and straight profiles). The post hoc analysis for multiple correction comparisons using the Tukey-Kramer method revealed that the difference was significant between concave and straight groups (t = 0.0139, *P* = 0.001) and also convex and straight groups (t = 0.0164, *P* = 0.0005), with no significant difference between concave and convex groups (t = -0.027, *P* = 0.97). According to Figure 2, the lowest excess cement was obtained using the straight emergence profile (10.47 ± 11.03 mg for the straight profile, 49.13 ± 35.83 mg for the concave profile, and 51.58 ± 49.34 mg for the convex profile).

**Figure 3:**
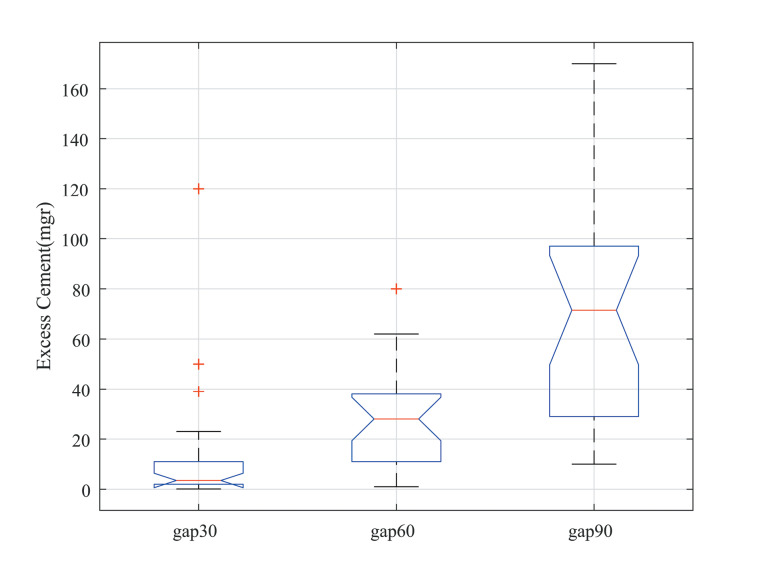
Average excess cement (mg) for different space gap sizes

 Considering the space gap size, the statistical analysis using one-way ANOVA revealed a significant difference between different groups. The post hoc multiple comparison correction tests showed a significant difference between gap 30-µm and 90-µm gaps (t = -0.079, *P* < 0.001) and 60-µm and 90-µm gaps (t = -0.066, *P* < 0.001), with no significant difference between 30-µm and gap 6- µm gaps (t = -0.036, *P* = 0.31). The average excess cement for the 30-µm gap group was 13.43 ± 25.85 mg, with 27.04 ± 19.7 mg for the 60-µm gap and 70.71 ± 45 mg for the 90-µm gap (Figure 3).

**Figure 4:**
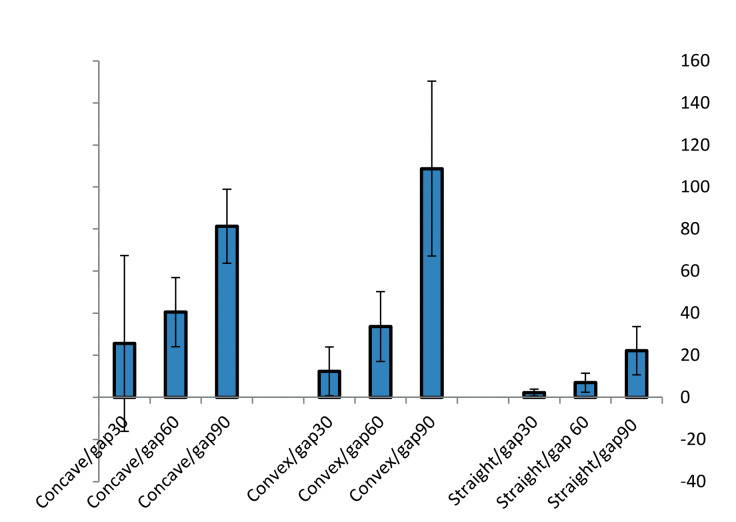
Comparison between excess cement for different emergence profiles and space gap sizes. The chart shows values using means ± standard deviations (n = 8 samples, mg)

 The statistical analysis for the interaction between two confounding factors (emergence profile and space gap size) was performed using two-way ANOVA. This analysis indicated a significant interaction between the emergence profile and gap size (F_(2,71)_ = 6.35, *P* < 0.001). Figure 4 shows the average value (n = 8) for excess cement (mg) for different emergence profiles and gap sizes.

**Table 1:** Excess cement (gr) values for eight different samples

**Sample no.**	**Concave gap 30**	**Concave** **gap 60**	**Concave** **gap 90**	**Convex** **gap 30**	**Convex** **gap 60**	**Convex** **gap 90**	***Straight*** **gap 30**	***Straight*** **gap 60**	***Straight*** **gap 90**
1	0.002	0.032	0.093	0.01	0.015	0.06	0.002	0.01	0.01
2	0.005	0.035	0.074	0.015	0.023	0.15	0.0001	0.012	0.023
3	0.023	0.036	0.066	0.012	0.026	0.1	0.002	0.005	0.025
4	0.001	0.031	0.069	0.003	0.042	0.078	0.003	0.001	0.026
5	0.002	0.08	0.1	0.008	0.019	0.15	0.001	0.012	0.032
6	0.002	0.03	0.054	0.039	0.062	0.17	0.0012	0.004	0.041
7	0.05	0.04	0.096	0.01	0.051	0.084	0.005	0.002	0.01
8	0.12	0.04	0.098	0.002	0.031	0.078	0.004	0.01	0.01

**Table 2:** Statistical analyses and effect sizes (Hedge’s g) for excess cement obtained for different conditions

	**Concave gap 30**	**Concave** **gap 60**	**Concave** **gap 90**	**Convex** **gap 30**	**Convex** **gap 60**	**Convex** **gap 90**	***Straight*** **gap 30**	***Straight*** **gap 60**	***Straight*** **gap 90**
KS normality test (P-value)	0.022	0.018	0.015	0.022	0.02	0.014	0.023	*0.022*	*0.021*
Hedge’s g effect size									
Concave gap 30	0	-0.44	-1.64	0.41	-0.24	-1.89	0.75	0.59	0.11
Concave gap 60		0	-2.27	1.87	0.39	-2.04	3.10	2.63	1.23
Concave gap 90			0	4.37	2.64	-0.81	5.98	5.47	3.77
Convex gap 30				0	-1.41	-2.98	1.15	0.58	-0.80
Convex gap 60					0	-2.24	2.52	2.08	0.76
Convex gap 90						0	3.42	3.25	2.68
*Straight *gap 30							*0*	-1.32	-2.29
*Straight *gap 60								*0*	-1.64
*Straight *gap 90									*0*

## Discussion

 In most previous studies, only convex and concave types of emergence profiles have been evaluated and compared for implantation. However, the current study considered the straight type of emergence profiles, which has been considered in recent studies. The results of the current study revealed thatthe straight type of emergence profile was associated with significantly (*P* < 0.05) lower excess cement compared with convex or concave types. Furthermore, there was no significant difference between convex and concave emergence profile designs. Our results are consistent with some studies and contradict some other studies. Sancho-Puchades et al^20^ reported that the concave emergence profile increased the risk of excess cement compared with the convex type (*P* = 0.043, according to non-parametric tests). However, according to Croll,^24^ selecting a straight emergence profile for artificial crowns improved hygiene effectiveness in the gingival sulcus. It seems that a straight profile might be more similar to the axial profile of teeth. According to the results of this study, convex or concave emergence profiles increased the risk of excess cement. Furthermore, convexity or concavity of a restoration may also trap plaque and disrupt the gingiva.^25^

 In addition, the results of our study indicated that increasing the gap size increased the risk of excess cement on the implant or teeth surface. It is clear from Figure 4 that for all three kinds of emergence profiles, such increased risk occurred. However, post hoc statistical analyses showed that the difference was significant when the gap size increased more than 60 µm (i.e., there was no significant difference between 30- and 60-µm gaps). Increasing the gap size from 30 µm to 60 µm increased the excess cement by 101.34% (from 13.43 ± 25.85 mg to 27.04 ± 19.7 mg), and increasing the gap size from 30 µm to 90 µm increased the excess cement by 426.5% (from 13.43 ± 25.85 mg to 70.71 ± 45 mg). The percentage of cement increment for increasing the gap size from 60 to 90 µm was 161.5% (from 27.04 ± 19.7 mg to 70.71 ± 45 mg). Previous studies confirmed the effect of gap size on marginal gap^26^ and marginal discrepancy and retention.^27^

 Statistical analyses using two-way ANOVA indicated a significant interaction between gap size and emergence profile (*P* < 0.05). According to Figure 4, for all emergence profiles, the excess cement increased gradually by increasing the gap size. However, for a 90-µm gap size, the excess cement increased considerably. Furthermore, for small gap size (i.e., 30 µm), a linear decrease in excess cement was observed when the emergence profile changed from concave to convex and straight. However, for larger gap size values, the change was nonlinear. One limitation of our study was related to using a fixed degree for convexity or concavity of the emergence profile. The degree of convexity (concavity) may affect the excess cement, which needs to be tested in future studies.

## Conclusion

 Excess cement in cement-retained implant reconstructions is one of the important aspects of implant dentistry.It directly affects the inflammation in the implant’s surrounding area and orodental health. This study evaluated the effect of the emergence profile and the abutment gap size on the excess cement. In brief, the straight emergence profile design could be the optimal design for reducing excess cement compared with concave and convex profiles. Furthermore, increasing the gap size increased the excess cement; however, it was significant if the gap size increased over a threshold. For design optimization, more research is needed to focus on the different angles of the emergence profile and different gap size values.

## Competing Interests

 There was nothing to declare.

## Consent for Participation

 None.

## Data Availability Statement

 Not applicable.

## Ethical Approval

 All procedures were followed by the revised version of the Helsinki Declaration, and the institutional review board approved all study aspects (IR.UMSHA.REC.1400.137).
